# Isolation and Identification of a New Phenylpropanoid Derivative from the Fruits of *Pimpinella Haussknechtii* Rech. f. & Riedl and GC-MS Analysis of the Essential Oil

**Published:** 2015

**Authors:** Seyed Ebrahim Sajjadi, Mustafa Ghanadian, Shahla Ahmadi, Sorour Shafiyan

**Affiliations:** a *Department of Pharmacognosy, School of Pharmacy, Isfahan University of Medical Sciences, Isfahan, Iran. *; b *Isfahan Pharmaceutical Sciences Research Center, Isfahan University of Medical Sciences, Isfahan, Iran.*; c *Lorestan Agriculture and Natural Resources Research Center, Khoramabad, Iran. *

**Keywords:** *Pimpinella haussknechtii*, Umbelliferae, 4-(prop-2-enyl)-phenyl-3'-methylbutyrate, Essential oil composition, Bicyclogermacrene

## Abstract

The chemical composition of the essential oil of fruits of *Pimpinella haussknechtii*. was studied by GC-MS. After GC-MS analysis, one unknown component (56.7%) was observed, which was not characterized in the GC-MS library. The essential oil of P.haussknechtii was injected to HPLC using YMC-Pak-Sil column (250 × 20 mm) with gradient system of hexane (A), and hexane: ethyl acetate, 9:1 (B) to yield the interested compound as a new phenylpropanoid derivative. Its structure was elucidated as 4-(prop-2-enyl)-phenyl-3'-methylbutanoate based on ^13^C- and ^1^H-NMR as well as 2D-NMR, IR and different MS spectra. In the essential oil analysis, thirty-six components, comprising 94.9% of the total oil, were identified. 4-(2-propenyl)-phenyl 3'-methylbutyrate (56.7%), bicyclogermacrene (8.9%), germacrene D (7.6%), perilla aldehyde (3.5%) and *β*-caryophyllene (2.9%) were found to be the major constituents of the oil. The oil of the fruits of *P. haussknechtii *consisted of eight monoterpene hydrocarbons (1.7%), two oxygenated monoterpenes (3.9%), sixteen sesquiterpene hydrocarbons (26.8%), two oxygenated sesquiterpenes (2.1%) and five phenylpropanoids (58.7%). Three other nonterpenic compounds also comprised 1.7% of the oil.

## Introduction

Umbelliferae family contains about 300 genera and 2500-3000 species distributed all around the world (1). The genus *Pimpinella *is one of the main genera of Umbelliferae and comprises more than 150 species (2). The genus represents in the flora of Iran by twenty species including six endemics (3). Previous phytochemical studies of *Pimpinella* species have led to the isolation of various compounds like phenylpropanoids (4) sesquiterpenes (5) coumarins (6) and volatile oils (7). According to pharmacological studies, the fruit of Anis* (Pimpinella anisum)* is widely used as carminative, expectorant and spasmolytic (8). It is also proved to possess antioxidant, antimicrobial, gastroprotective, antifungal, anticancer and bronchodilatory activities (9-14). It is used in traditional medicine for *menopausal hot flashes (*14*).* *In-vitro* study of *P. brachycarpa*, edible greens grown in Asian regions, is found to have antioxidant effects (15). *P. anisoides* inhibits acetylcholinesterase (16) and presents protective effect on oxidative damages (17). *P. tirupatiensis* have also shown cardio-protective activity on doxorubicin induced cardiotoxicity in rats (18). Economically, this genera are cultivated all around the world as medicinal plant. A few other species are cultivated for their aromatic fruits such as *P. anisetum *in Russia and *P. saxifraga *in India. *P. peregrina *and *P. major *are cultivated in Germany for their roots and *P. calycina *as vegetable (18). *Pimpinella haussknechtii* Rech. f. & Riedl (Syn. *P.* *kotschyana* Boiss.) (20) is an annual native plant which grows in the west of Iran. 

Available information indicates that flavonoids and essential oils are two secondary metabolites which have been reported from different parts of *P.* *kotschyana* (22, 21).

There are also a report on chemical composition and antimicrobial activity of *P.* *kotschyana* oil collected from Tehran province, Iran (23).

In this study, the volatile oil constituents of the friuts of *P. haussknechtii *grown in Lorestan province, Iran is reported by using the GC-MS analysis for the known components and high pressure liquid chromatography for unknown compound.

## Experimental

*General*

HPLC (High-performance liquid chromatographic) analysis was done on a Waters system, equipped with 515 HPLC pump, and waters 2487 dual wavelenghth absorbance detector (Waters, Milford, MA, USA). The column was a YMC-Pak SIL (250 × 20 mm) (YMC Europe GmbH, Germany). The NMR spectra were recorded on a Bruker Avance AV 400 instrument, using CDCl_3_ as a solvent. The IR spectrum was recorded on a Rayleigh WQF-510 FTIR spectrophotometer and the HREI-MS spectrum was measured in electron impact mode on Varian MAT 312 spectrometer.

*Plant material*

The fruits of *P. haussknechtii *were collected during July 2012 from Khoramabad in the west of Iran at an altitude of *ca.* 1100 m above sea level. The plant was identified by Khoramabad Agricultural and Natural Resource Research Center. A voucher specimen (No 2827) was deposited at the Herbarium of the School of Pharmacy and Pharmaceutical Sciences, Isfahan University of Medical Sciences, Isfahan, Iran.

*Extraction and isolation*

The essential oil of the fruits of *P. haussknechtii *was obtained by hydrodistillation using a Clevenger-type apparatus for 3 h according to the method recommended by the British Pharmacopoeia (24). The volatile oil was dried over anhydrous sodium sulfate and stored in sealed vial at 4°C until analysis. Gas chromatography combined with mass spectrometry was used for identification of the known oil components. Firstly, the analysis was performed on an Agilent 5975C mass selective detector coupled with an Agilent 7890A GC, equipped with an HP-5 GC capillary column (30 m × 0.25 mm; film thickness 0.25 μm). The oven temperature was programmed from 60-280°C at the rate of 4°C per min. Helium was used as the carrier gas at a flow rate of 2 mL/min. Injector and detector temperatures were 280°C. The MS operating parameters were: ionization voltage, 70 eV; ion source temperature, 230°C; mass range, 35-425. The MSD ChemStation was used as operating software. Retention indices were calculated by using retention times of *n*-alkanes (C_8_-C_24_) that were injected after the oil at the same conditions. Components of the oil were identified by comparison of their retention indices (RI) with those reported in the literature (21) and computer matching with NIST and Wiley275. L libraries. The fragmentation patterns of the mass spectra were also compared with those reported in the literature (25, 27).

After GC-MS analysis, one unknown component (56.7%) was observed with retention time of 22.8 min, not characterized in the GC-MS library. In order to identify this compound, the essential oil was subjected on HPLC using YMC-Pak-Sil column (250 × 20 mm) with gradient system of hexane (A), and hexane: ethyl acetate, 9:1 (B) starting with A: B (100: 0) for 20 min, then 0−20% B in 50 min, A:B (80: 20) for 50 min, then 20-30% B in 30 min, and 30-100% B for 50 min. The flow rate was 3 mL/min, UV ditection at 210 and 270 nm , and the injection volume was 100 μL. The composition of each fraction was controlled by GC/MS analysis and the HPLC retention time for the compound of interest was found to be 116-122 min.

4-(prop-2-enyl)-phenyl-3'-methylbutanoate (1). White solid; [*α*]_D:_ −20.4 (c 0.18, CDCl_3_); IR (KBr) ν_max_:3080, 3005, 2962, 2933, 2873, 1759, 1639, 1608, 1506, 1468, 1435, 1417, 1369, 1292, 1203, 1165, 1101, 1018, 995, 916, 850, 771 cm^-1^; ^1^H-NMR (CDCl_3_, 400 MHz, *J *in Hz) and ^13^C-NMR (CDCl_3_, 100 MHz) see Table 1. HREI-MS *m/z* 218.1296 (calc. for C_14_H_18_O_2_, 218.1307, Δ -4.8 ppm), Positive EI-MS *m/z *218 (10), 134 (100), 119 (12), 115 (15), 107 (20), 105 (11), 103 (7) , 91 (16), 85 (22), 77 (23), 57 (40).

**Table 1:** ^1^H- and ^13^C-NMR data for 4-(prop-2-enyl)-phenyl-3'-methylbtyrate

Pos	δ_H_, mult., *J i*n Hz	δ_C_	Pos	δ_H_, mult., *J* in Hz	δ_C_
1	-	171.7	*O*-3'-MB		
2	6.91 d (8.4)	121.5	1'	-	171.7
3	7.12 d (8.4)	129.5	2'	2.35, d (7.2)	43.4
4	-	140.4	3'	2.18, m	25.9
5	7.12 d (8.4)	137.5	4'	0.98, d (6.8)	22.4
6	6.91 d (8.4)	121.5	5'	0.98, d (6.8)	22.4
1''	3.30, d (6.4)	39.6			
2''	5.88, m	137.2			
3''a	5.03, dd (18.4, 1.8)	116.0			
3''b	5.01, dd (10.8, 1.8)				

## Results and Discussion

More than thirty-six components were detected in the fruits of *P. haussknechtii* (Table 2). Thirty-five components of the oil were identified by GC-MS method and then HPLC method was used for the isolation of one unknown component (56.7%), which was not characterized in the GC-MS library with retention time of 22.8 min. 

**Table 2:** Composition of the essential oil of the fruits of *Pimpinella haussknechtii*

**No**	**Compound**	**RT**	**RI Calc.** a	**RI** b	**%** c	**Identification** **Method**
1	α-pinene	3.79	940	939	0.1	RI, EI-MS
2	camphene	4.06	950	953	t^c^	RI, EI-MS
3	sabinene	4.52	973	975	t	RI, EI-MS
4	β-pinene	4.61	978	979	1.1	RI, EI-MS
5	myrcene	4.86	990	991	0.1	RI, EI-MS
6	limonene	5.74	1029	1029	0.3	RI, EI-MS
7	γ-terpinene	6.48	1059	1062	t	RI, EI-MS
8	*m*-cresol	6.95	1076	1077	0.4	RI, EI-MS
9	terpinolene	7.37	1087	1088	0.1	RI, EI-MS
10	nonanal	7.72	1102	1102	0.1	RI, EI-MS
11	ethyldimethylthiophene	9.69	1170	-	1.2	EI-MS
12	methyl chavicol	10.51	1195	1195	0.2	RI, EI-MS
13	chavicol	12.3	1254	1253	1.4	RI, EI-MS
14	perilla aldehyde	12.64	1268	1272	3.5	RI, EI-MS
15	bornyl acetate	13.25	1283	1285	0.4	RI, EI-MS
16	α-ylangene	16	1371	1372	0.3	RI, EI-MS
17	β-elemene	16.53	1387	1391	0.4	RI, EI-MS
18	cyperene	16.72	1394	1398	0.5	RI, EI-MS
19	methyl eugenol	16.97	1401	1401	0.1	RI, EI-MS
20	β-caryophyllene	17.35	1414	1418	2.9	RI, EI-MS
21	aromadendrene	18.23	1444	1441	0.4	RI, EI-MS
22	α-humulene	18.35	1449	1454	0.2	RI, EI-MS
23	*trans*-β-farnesene	18.56	1455	1458	0.8	RI, EI-MS
24	drima-7,9(11)-diene	19.11	1473	1473	1.5	RI, EI-MS
25	germacrene D	19.27	1482	1485	7.6	RI, EI-MS
26	β-selinene	19.53	1486	1490	1.6	RI, EI-MS
27	bicyclogermacrene	19.75	1495	1496	8.9	RI, EI-MS
28	α-selinene	19.94	1499	1498	0.7	RI, EI-MS
29	cis-α-bisabolene	20.05	1503	1504	t	RI, EI-MS
30	γ-cadinene	20.27	1511	1514	0.2	RI, EI-MS
31	*cis*-γ- bisabolene	20.39	1516	1515	0.3	RI, EI-MS
32	*trans*-γ- bisabolene	21.05	1539	1531	0.5	RI, EI-MS
33	spathulenol	22.02	1572	1576	0.9	RI, EI-MS
34	new compound (cmpd. **1**)^e^	22.88	1601	-	56.7	NMR, HREI-MS
35	foeniculin	24.8	1673	1678	0.3	RI, EI-MS
36	*cis*-γ-atlantone	25.11	1689	1694	1.2	RI, EI-MS

a RI= Calculated retention indices on HP-5 GC capillary column;

b RI= Reference retention indices;

c Percentages calculated from TIC data;

d t = trace (<0.05%);

e 4-(prop-2-enyl)-phenyl-3'-methylbutyrate.

**Figure 1:**
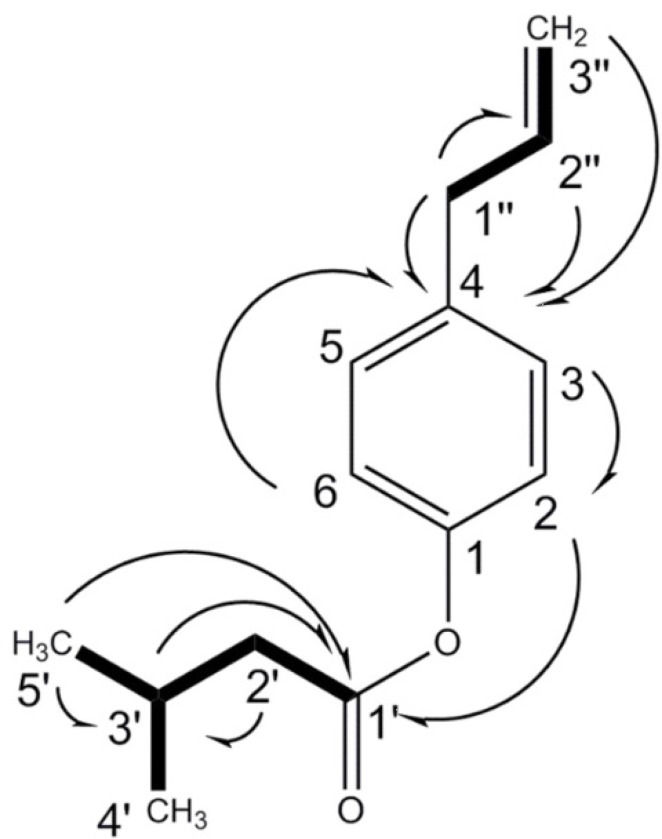
Key ^1^H-^1^H COSY (in bold) and HMBC correlations ( ) of 4-(prop-2-enyl)-phenyl-3'-methylbutyrate

**Figure 2:**
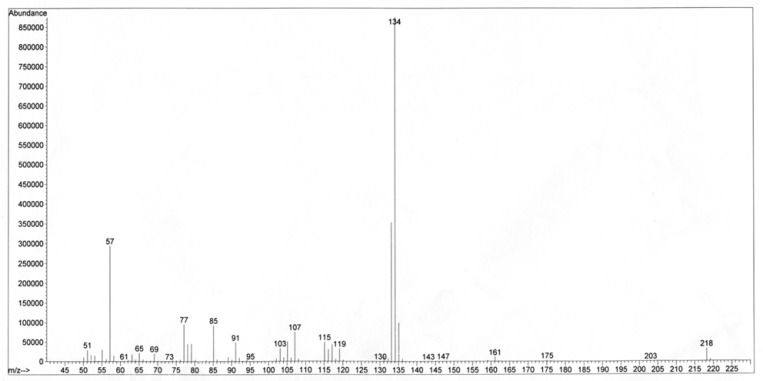
Mass spectrum of 4-(prop-2-enyl)-phenyl-3'-methylbutyrate; RI=1601 on HP-5 GC column.

The unknown compound was assigned the molecular formula C_14_H_18_O_2_ based on HREI-MS positive mode *m/z* 218.1296 (calc. for C_14_H_18_O_2_, 218.1307, Δ -4.8 ppm), in agreement with the number of carbons and hydrogens in the NMR spectra (Table 1). The IR absorptions indicated the peaks of carbonyl (1759 cm^-1^), C-O (1203-1101 cm^-1^), aromatic or olefinic bonds (3080, 1639, and 1506), with no free hydroxyl group. The ^13^C NMR spectrum supported the existence of the aromatic ring showing six carbon peaks δ_C_ 149.0 (s, C-1), 121.5 (d, C-2, C-6), 129.5 (d, C-3, C-5), and 137.2 (s, C-4) at the aromatic region. The carbon resonances were assigned by the use of HSQC spectrum. The ^1^H-NMR spectrum showed AA'XX' spin pattern of *p*-disubstiuted aromatic rings for H-2, 6, and H-3, 5 at δ_H_ 6.92 (2 × H, d, *J* = 8.4 Hz, A, A' of AA'XX'), and 7.11 (2 × H, d, *J* = 8.4 Hz, X, X' of AA'XX'), respectively. In addition, NMR signals indicated the presence of 3'-methyl butanoyl moiety at δ_C_ 171.7, 43.4 (δ_H_ 2.35 d, *J* = 7.2, 2 × H); 25.9 (δ_H_ 2.18 m, 1 × H); 22.4 (δ_H_ 0.98 d, *J* = 6.8 Hz, 2 × 3H) and an allyl group at δ_C_ 39.6 (δ_H_ 3.30 d, *J* = 6.4, 2 × H, H-1''); 137.2 (δ_H_ 5.88 m, 1 × H, H-2''); 116.0 (δ_H_ 5.03 dd, *J* = 18.4, 1.81Hz, H-3''a/ 5.01 dd, *J* = 10.8, 1.81Hz, H-3''b). ^1^H-^1^H COSY, as well as HMBC correlations (Figure 1), confirmed the coupling between the protons and characteristic connectivities of the sidechain C1''-H (δ_H_ 3.30) with C-4 (δ_C_ 137.2) of the aromatic ring. The structure was also confirmed through EI mass ion fragments at *m/z* 218 [M], 134, 133 [238-3'MB], 91 [M-3'MB –allyl], 77 [C6H5] (Figure 2), which allowed us to establish the structure as 4-(prop-2-enyl)-phenyl-3'-methylbutyrate. Literature survey revealed that 4-(prop-2-enyl)-phenyl-3'-methylbutyrate is a new compound reported for first time. 4-(Prop-2-enyl)-phenyl angelate, its similar compound differed in the type of ester attached to the phenyl ring, was previously reported from essential oil of fruits of *P. isaurica* (5).

Taken together, GC Mass analysis and HPLC analysis showed that the oil of the fruits of *P. haussknechtii *consisted of eight monoterpene hydrocarbons (1.7%), two oxygenated monoterpenes (3.9%), sixteen sesquiterpene hydrocarbons (26.8%), two oxygenated sesquiterpenes (2.1%) and five phenylpropanoids (58.7%). Three other nonterpenic compounds were also consisted 1.7% of the oil. The identified components are listed in order of their elution on the HP-5 GC column (Table 2). 4-(2-Propenyl)-phenyl 3'-methylbutyrate (56.7%), bicyclogermacrene (8.9%), germacrene D (7.6%), perilla aldehyde (3.5%), and *β*-caryophyllene (2.9%) are the main constituents of the oil.

According to the previous study on essential oil composition of fruits of *P. kotschyana* grown in Tehran, *β*-caryophyllene (40.6%), germacren D (11.3%), langipinalol (17.6%) and limonene (7.8%) were the major constituents of the oil (D). The main componenets of fruits of *P. kotschyana* gathered from central parts of Turkey were also reported as β-caryophyllene (49.3%), *α*-humulene (11.0%), 12-hydroxy-β-caryophyllene acetate (11.5%) and caryophyllene oxide (3.0%) (B). In contrast, 4-(prop-2-enyl)-phenyl-3'-methylbtyrate (56.7%), and bicyclogermacrene (8.9%) were the main component of the *P. haussknechtii* fruit oil collected from Lorestan, Iran and *β*-caryophyllene was present in the minor amounts (2.9%).

Phenylpropanoids found in high contents in the oil of different *Pimpinella *species, were classified in two groups of propenylphenol-type (4-monosubstituted phenylpropanoid) and pseudoisoeugenol-type (2,5-disubstituted phenylpropanoid) (5) from which 4-(prop-2-enyl)-phenyl-3'-methylbutyrate belongs to first group. Previous studies on the volatile oil of fruits of members of *Pimpinella* genus showed various compositions. *trans*-Anethole is the major component (75-95%) of *P. anisum* which could be affected by the genotype and ecological conditions (22-24). Limonene is reported as the major components of* P. affinis *(90.5%), *P. puberula* (82.4%) and *P. eriocarpa* (49.3%) (26,28). β-Pregeijerene (87.0%), bisabolene (50.8%), β-pinene (25.3%) and methyl eugenol (18.7%) are also reported as the major constituents of the essential oils of *P. tragioides*, *P. aurea, P. tragium *and *P. barbata*, respectively (7, 34-36). 

The fruits of *P. haussknechtii *yielded 1.5% (v/w) of yellowish oil with an aromatic odor. Essential oil yields of fruits of different *Pimpinella* species are very variable, for example the volatile oil yields of fruits of *P. cretica *var.* arabica *and* P. isaurica* are 10.0% and 0.3%, respectively. There are also other species that their fruits have no volatile oil (5).
